# Developing an information literacy competency evaluation framework for Chinese higher vocational students in smart older adult care: a Delphi-AHP study

**DOI:** 10.3389/fpubh.2026.1906092

**Published:** 2026-07-29

**Authors:** Fangfang Du, Yajuan Zhou, Chaojin Da, Kun Zhang, Feifan Liu, Feifei Wan, Yujing Dong

**Affiliations:** 1 Henan Vocational College of Tuina, Luoyang, China; 2 Faculty of Education, Beijing Normal University, Beijing, China; 3 School of Nursing and Rehabilitation, Shandong University, Jinan, China; 4 School of Nursing, Nanjing University of Chinese Medicine, Nanjing, China

**Keywords:** analytic hierarchy process, Delphi method, evaluation framework, information literacy, smart older adult care, vocational education

## Abstract

**Background:**

The integration of digital technologies into older adult care requires higher vocational students majoring in Smart Older Adult Care to possess information literacy. However, higher vocational education cultivates technical and skilled talents, while undergraduate education focuses on academic research-oriented talents; thus, their information literacy frameworks cannot be interchanged. Yet prior research has largely focused on general university students or healthcare professionals, lacking targeted evaluation tools for higher vocational students. The gap impedes evidence-based teaching assessment and targeted talent development.

**Objective:**

To develop an information literacy evaluation framework for higher vocational students majoring in Smart Older Adult Care and calculate the weights of its indicators.

**Design:**

A Delphi–AHP study.

**Methods:**

A multi-method study was conducted in China (April–October 2025), integrating a systematic literature review, expert group discussions, a two-round Delphi survey involving 17 experts, and the Analytic Hierarchy Process (AHP).

**Results:**

Expert positivity coefficients were 94.4% in the first round and 100% in the second, with an authority coefficient of 0.841. Kendall’s *W* were 0.217 and 0.246 (both *p* < 0.001). The final framework consisted of 4 first-level, 13 s-level, and 44 third-level indicators, with all consistency ratios (*CR*) < 0.1. The first-level weights were as follows: Information Application and Innovation (0.4718), Information Awareness (0.3073), Information Knowledge (0.1228), and Information Ethics and Security (0.0981).

**Conclusion:**

This study developed a preliminary information literacy framework tailored to higher vocational students, emphasizing practical competence. Further psychometric verification is recommended.

## 1 Introduction

Population aging represents a defining global trend, and China is experiencing the world’s largest and most rapidly growing older adult population (1). In response, the integration of the Internet of Things (IoT), big data, and artificial intelligence into older adult care services has positioned smart older adult care as a key approach in modern healthcare systems (2). This demographic shift has placed new demands on older adult care workforce, which requires both more personnel and higher competence (3). Professionals in smart older adult care are expected to possess information literacy—that is, the ability to recognize information needs, locate and critically evaluate relevant information, and apply it effectively to problem-solving. Such literacy enables practitioners to navigate digital devices, interpret health data, coordinate care through digital platforms, and ultimately deliver technology-enhanced, person-centered services to older adults (4).

In the past two decades, information literacy has been extensively conceptualized through several foundational frameworks. The Association of College and Research Libraries (ACRL) Framework highlights threshold concepts such as “scholarship as conversation” and “research as inquiry” (5). The Australian and New Zealand Information Literacy Framework (ANZIIL) delineates six core standards encompassing information needs identification, access, evaluation, management, and ethical use (6). Similarly, the Society of College, National and University Libraries (SCONUL) Seven Pillars model outlines a developmental pathway from identifying information needs to organizing and presenting information (7). Across these frameworks, common dimensions can be distilled: information awareness, knowledge, capability, and ethics (8).

Building on these generic models, there is growing recognition of the need for discipline-specific adaptations. In nursing education, Groom et al. (9) developed an information literacy evaluation tool for undergraduate nursing students comprising four dimensions: information awareness, information retrieval, information evaluation, and information application. Fei et al. (10) further validated a five-factor model that adds ethical and security considerations. For clinical nurses, Lee et al. (11) proposed a framework emphasizing evidence-based practice skills and clinical decision-making using digital resources. Similarly, studies focusing on healthcare professionals, social rescue personnel, and teacher education students (11–13) have consistently shown that effective information literacy assessment must account for the unique knowledge domains, practice contexts, and competency expectations of each profession. For digital literacy among vocational students in older adult care, Guo et al. (14) proposed a preliminary framework encompassing digital skills, knowledge, awareness, attitudes, and values. However, this framework remains at a conceptual stage without empirical validation or indicator weighting. Collectively, these discipline-specific efforts provide a solid foundation for the current study. They confirmed that tailored frameworks are feasible, provide validated dimensions for adaptation, and highlight a critical gap: the current lack of a rigorously developed and weighted information literacy evaluation tool specifically for higher vocational students in smart older adult care.

Higher vocational education differs fundamentally from general higher education, emphasizing technical and applied competencies tied directly to industry needs (15). In 2021, the Ministry of Education of the People’s Republic of China formally restructured the “Older Adult Service and Management” program into “Smart Health and Older Adult Care Services and Management” (hereinafter “Smart Older Adult Care”). The Ministry explicitly stated that graduates should be equipped not only with traditional care knowledge but also with the ability to apply modern information technologies to complex real-world problems. This vocational discipline prepares service providers and care managers for a range of older adult care settings. In this respect, it is functionally distinct from gerontological nursing, which operates under separate competency standards and regulatory pathways. Given the increasing demands of this field, no empirically validated framework currently exists to assess the information literacy of this specific student group. Its absence not only prevents evidence-based teaching evaluation and hampers targeted talent development, but also restricts educators in assessing how well curricular interventions respond to rapid technological change.

To address this gap, the study set out to develop an information literacy evaluation framework specifically for higher vocational students in the Smart Older Adult Care, and to assign relative weights of its indicators. This resulting framework is intended to support both the assessment of students’ information literacy and the ongoing development of related curricula. Ultimately, it seeks to prepare a tech-proficient workforce for older adult care.

## 2 Methods

### 2.1 Study design

**Figure 1:**
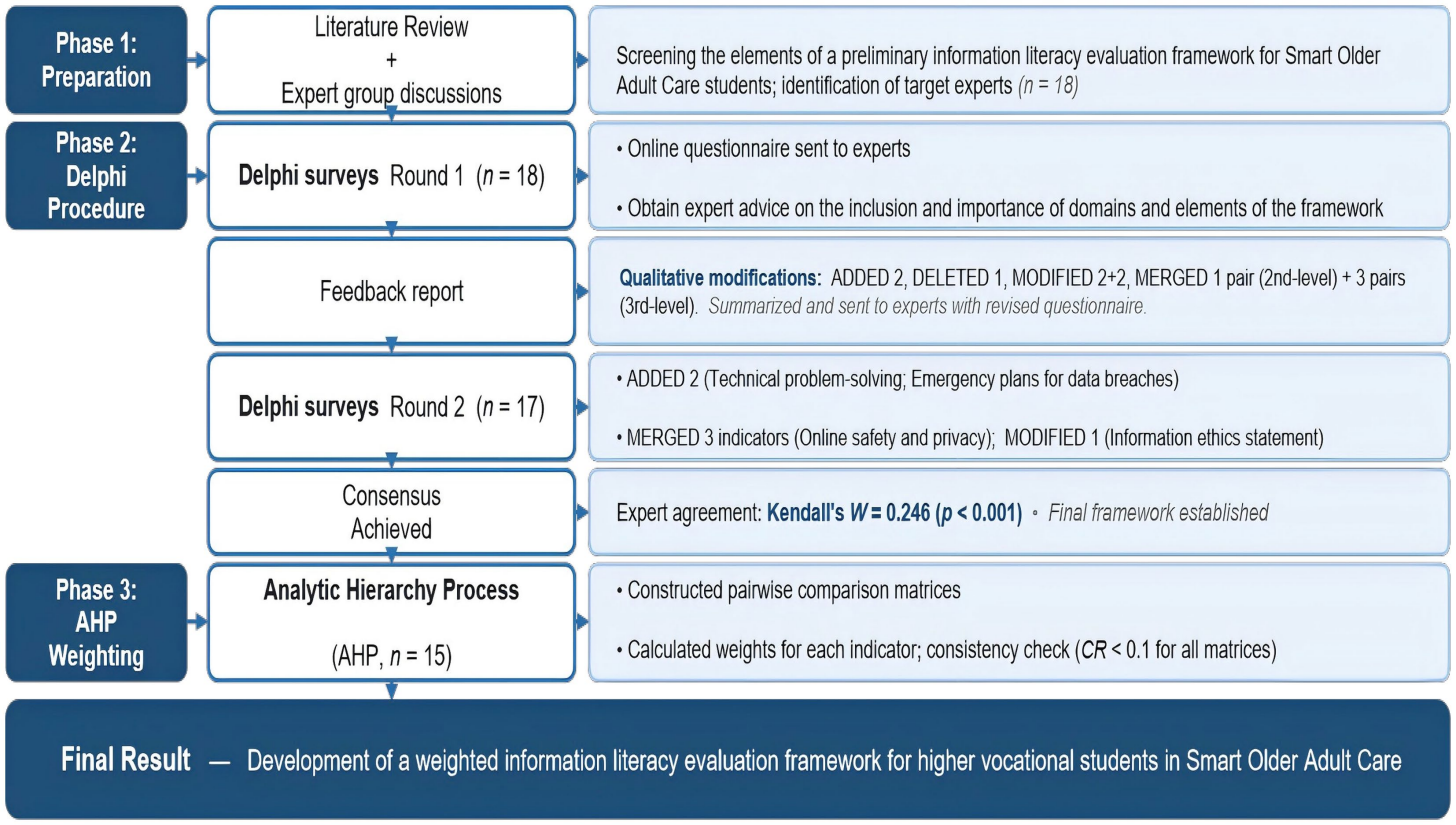
Overview of the study design and methodology.

A multi-method study was conducted in three sequential phases to ensure the scientific rigor of the evaluation framework development as described in Figure 1. Phase 1 consisted of developing a preliminary evaluation framework through systematic literature review and expert group discussions. Phase 2 involved a two-round Delphi survey to establish expert consensus on the indicators and their relevance. Phase 3 applied the Analytic Hierarchy Process (AHP) to determine the weight of each indicator at all levels. The entire study process was guided by the Delphi Critical Appraisal Tool (DCAT) (16) to ensure methodological rigor.

#### 2.1.1 Phase 1: literature review and expert discussion

**Figure 2:**
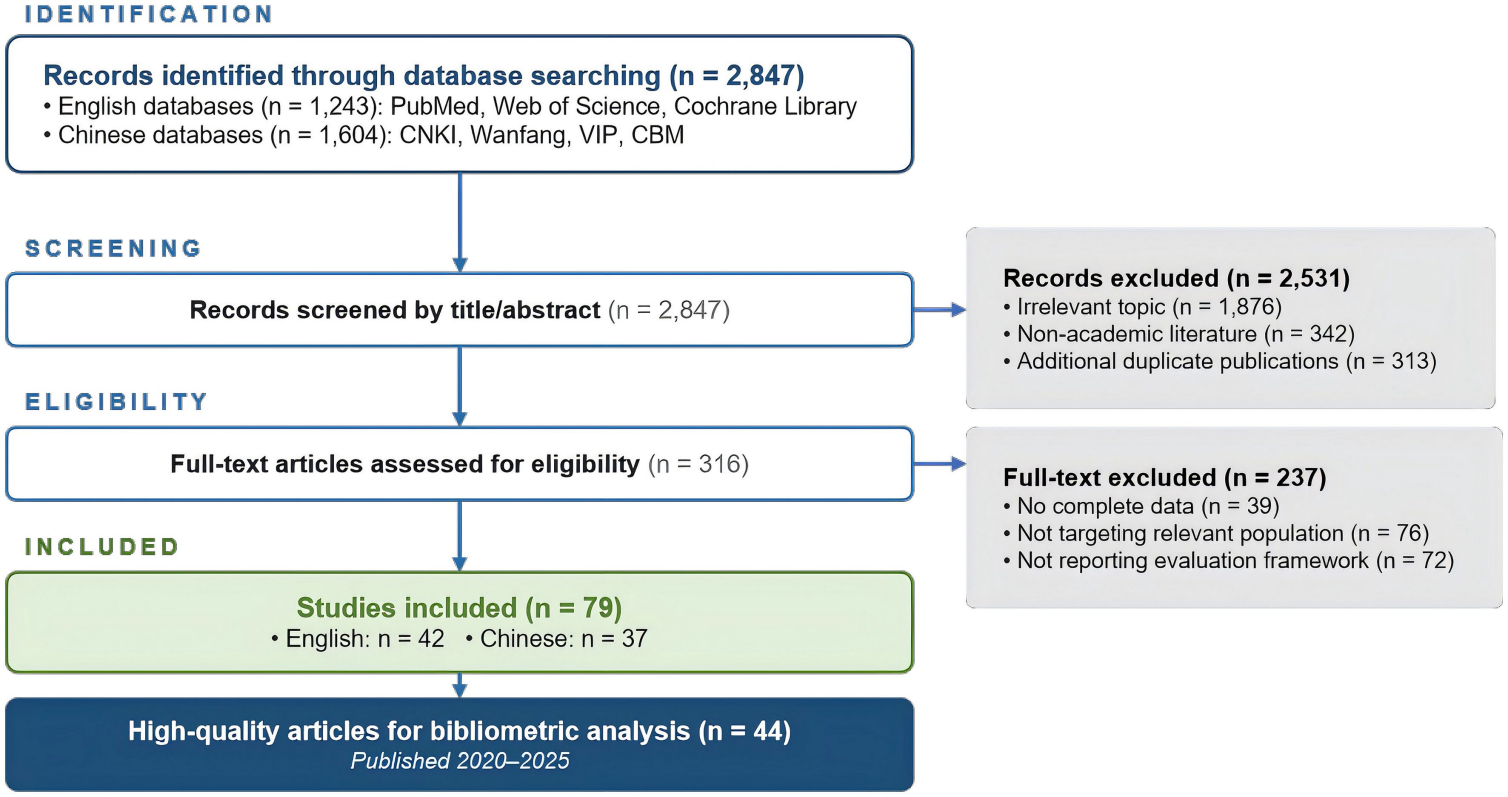
PRISMA flow diagram.

A systematic literature search was conducted in the Cochrane Library, PubMed, Web of Science, VIP, CNKI, Wanfang, and the Chinese Biomedical Literature Database, covering the period from January 2015 to March 2025. The search strategy employed terms from three domains: (1) population and setting (e.g., “smart older adult care,” “smart geriatric care,” “older adult care,” “aged care,” “older adults*,” “vocational student*,” “polytechnic”); (2) the construct of interest (e.g., “information literacy,” “digital literacy,” “information competence*,” “digital competence*,” “information skill*”); and (3) outcomes (e.g., “evaluation framework*,” “assessment framework*,” “competency framework*,” “competency model*,” “evaluation index*,” “indicator system”). A total of 2,847 records were identified. After screening titles and abstracts, 316 full-text articles were assessed for eligibility, and 79 met the inclusion criteria (42 English, 37 Chinese). Inclusion criteria were limited to peer-reviewed articles reporting evaluation frameworks or indicators related to information literacy in health professions, older adult care, or vocational education. Exclusion criteria removed conference abstracts, non-empirical works, duplicates, and articles without full text. The PRISMA flow diagram (Figure 2) summarizes the screening process, including the specific reasons for exclusion at each step.

Two researchers independently extracted information literacy-related items from the 79 included articles using a line-by-line approach. Extracted items were recorded in a standardized form, producing 127 candidate indicators. These were refined by merging duplicates, grouping similar items thematically, and removing items irrelevant to the vocational older adult care context. The remaining indicators were categorized under four theoretical dimensions: information awareness, information knowledge, information application and innovation, and information ethics and security. Following this refinement, the 127 candidate indicators were reduced to 46 preliminary ones. Disagreements between reviewers were resolved through discussion; when consensus could not be reached, a third senior researcher adjudicated. Five experts from the four disciplinary domains then reviewed the draft framework for relevance, clarity, and comprehensiveness. Based on their feedback, indicators were reworded, merged, deleted, or added as appropriate. This resulting revised framework served as the basis for the two-round Delphi survey.

To complement the literature-derived indicators, relevant policies, professional standards, and existing information literacy frameworks were also reviewed. These included the Chinese national teaching standards for the Smart Older Adult Care program (17), the ACRL Framework (5), the ANZIIL Framework (6), and other related literature (8, 18). A bibliometric analysis was performed on a subset of 44 high-quality articles published between 2020 and 2025, selected from the 79 included studies, to identify frequently cited and emerging indicator elements. Based on the theoretical framework and the program’s talent cultivation objectives, the research team consolidated all inputs (literature-derived indicators, bibliometric findings, and policy documents) into a draft framework through multiple rounds of discussion. The draft comprised 4 first-level, 14 s-level, and 46 third-level indicators.

#### 2.1.2 Phase 2: Delphi survey

##### 2.1.2.1 Questionnaire development

The first-round Delphi questionnaire was developed based on the preliminary index. It consisted of three sections. The first provided an introduction to the study background, objectives, key concept definitions, and instructions. In the second section, experts rated the importance of each indicator on a 5-point Likert scale (1 = not important to 5 = very important) and were given space to comment, suggest modifications, or propose additions or deletions. The third section collected experts’ demographic data and self-assessed authority.

##### 2.1.2.2 Recruitment of the Delphi expert panel members

A Delphi survey involves multiple rounds of consultation using questionnaires to obtain the collective opinion of experts until a consensus is reached (18). A purposive sampling method was used to recruit experts for the panel, with the following inclusion criteria: (1) engaging in work related to older adult care, information technology, or vocational education assessment for at least 5 years; (2) holding an associate senior title or above (or equivalent professional qualification); (3) having a clear understanding of the smart older adult care industry and information literacy requirements for vocational students; (4) being willing to participate in the two-round Delphi survey and provide honest feedback. 18 experts from 9 Chinese provinces were invited, of whom 17 completed both rounds. The panel represented four disciplinary areas—nursing education, gerontological nursing, older adult care industry, and information technology —to ensure a multidisciplinary perspective. Experts with gerontological nursing backgrounds were included because their knowledge of information needs in older adult care settings is directly relevant to the practice contexts of Smart Older Adult Care graduates. All experts evaluated competencies specifically for Smart Older Adult Care vocational students.

The final panel of 17 respondents falls within the commonly recommended Delphi sample size of 10–20 for heterogeneous topics, which is consistent with the majority of published Delphi studies (19, 20). To minimize selection bias, we adopted several measures, including clearly defined eligibility criteria for purposive sampling, stratified recruitment with preset quotas across the four disciplinary domains, geographic representation from nine provinces, independent candidate screening by research team members prior to invitation, and comprehensive documentation of all recruitment decisions. Snowball sampling and personal referrals were not used.

##### 2.1.2.3 Delphi method implementation

The Delphi survey was conducted between July and October 2025. Questionnaires were distributed and returned via email, WeChat, or in person. After each round, data were analyzed. For item retention in the first round, the criteria were: mean importance score ≥ 3.50, coefficient of variation (*CV*) ≤ 0.25, and full-score rate ≥ 20% (20). Experts’ qualitative comments were also collated. The research team then collectively reviewed all feedback and modified the evaluation framework accordingly—adding, deleting, merging, or revising items as appropriate. A revised questionnaire was then sent to the same panel for the second round, along with a summary of the first-round results and the team’s responses to expert comments. The Delphi process was concluded when expert opinions converged, and the final evaluation framework was established. The entire process was conducted in accordance with the “iteration” and “controlled feedback” principles of the DCAT (16).

The Delphi process was concluded after two rounds. The proportion of experts providing new written comments decreased sharply from 76.47% in the first round to 17.65% in the second, while all indicators met the retention criteria and the coefficient of variation fell to ≤ 0.115 across all items. These findings suggested that expert ratings had stabilized and additional rounds would yield little further refinement.

#### 2.1.3 Phase 3 analytic hierarchy process

The Analytic Hierarchy Process (AHP) is a quantitative technique for multi-criteria decision analysis that uses pairwise comparisons and eigenvalue computation to derive relative weights of criteria and alternatives (21). Following the Delphi survey, the AHP was used to determine the weight of each indicator. First, a hierarchical model was constructed based on the final evaluation framework, with the target layer (information literacy evaluation framework), criterion layer (first-level indicators), sub-criterion layer (second-level indicators), and indicator layer (third-level indicators). Then, a judgment matrix was constructed based on experts’ pairwise comparisons of the relative importance of indicators at the same level using Saaty’s 9-point scale (1 = equally important, 9 = extremely more important).

Fifteen of the 17 Delphi experts participated in the weighting stage. Individual judgment matrices were aggregated using the geometric mean method to obtain an integrated matrix for each level, from which weights were derived from the integrated matrix using the characteristic root method. Consistency was assessed using the *CR* (consistency ratio) = *CI* / *RI*, where *CI* = (*λ*max − *n*)/(*n* − 1) and *RI* values were taken from Saaty’s standard table.

### 2.2 Ethical considerations

This Delphi study involved expert participants and therefore constituted human subjects research. Before collecting any data, we submitted the study protocol to the Medical Ethics Committee of Nanjing University of Chinese Medicine for ethical review. In April 2025, the committee determined that the study was exempt from full ethical review. Before administering the first Delphi questionnaire, every expert was told about the study’s purpose, the voluntary nature of participation, and the measures for keeping their feedback confidential. Written informed consent was obtained from each expert before taking part in the survey. Throughout the research we followed the three core biomedical ethical principles: respect for persons, beneficence, and justice.

All expert feedback and the scoring data from the Delphi survey and AHP were anonymized before analysis and used only for the present research. Confidentiality was strictly maintained throughout the study; no personally identifiable information of participants was collected, stored, or disclosed in any form.

### 2.3 Data analysis

Data were analyzed using SPSS 26.0 and Yaahp 12.6. Descriptive statistics, including the mean, standard deviation, frequency, and percentage were calculated. Expert positivity was assessed by the questionnaire response rate and the proportion of experts who provided comments. Expert authority was measured by the authority coefficient (*Cr*), calculated as *Cr* = (*Ca* + *Cs*)/2, where *Ca* is the coefficient of judgment basis and *Cs* is the coefficient of familiarity with the topic. Coordination of expert opinion was evaluated using the coefficient of variation (*CV*) for each indicator and Kendall’s coefficient of concordance (*W*) for all indicators, with their statistical significance tested by the chi-square test. A significance level of *α* = 0.05 was adopted for all analyzes.

The weight coefficient of each indicator was calculated using the geometric mean method. Consistency testing was performed to ensure the rationality of the weight distribution, with the consistency ratio (*CR*) as the evaluation standard. *CR* < 0.1 was considered acceptable, indicating that the judgment matrix had satisfactory consistency (21, 22).

## 3 Results

### 3.1 Expert panel demographics

A total of 17 experts from 9 provinces (Beijing, Shanghai, Guangdong, Shandong, Sichuan, Henan, Jiangsu, Gansu, Zhejiang) in China participated in both rounds of the Delphi survey, ensuring geographic representativeness. The expert panel included 2 males (11.76%) and 15 females (88.24%). Their mean age was 38.06 years (SD = 5.09), with a mean working experience of 14.00 years (SD = 5.00). Twelve experts (70.59%) held a master’s degree or higher, indicating high academic and professional background. Detailed demographic information is presented in Table 1.

**Table 1:** Demographic characteristics of the expert panel (*n* = 17).

Demographic characteristics		Frequency	Percentage(%)
Gender	Male	2	11.76
	Female	15	88.24
Age (years)	30–40	11	64.71
	>40	6	35.29
Education Level	Bachelor’s	5	29.41
	Master’s	10	58.82
	Doctoral	2	11.76
Professional Title	Mid-level	8	47.06
	Associate Senior	8	47.06
	Senior	1	5.88
Years of Experience	<10	4	23.53
	10–20	10	58.82
	20–30	3	17.65
Specialization	Nursing Education	7	41.18
	Gerontological Nursing	5	29.41
	Older Adult Care Industry	2	11.76
	Information Technology	3	17.65

Specialization categories reflect experts’ professional backgrounds. All experts evaluated literacy for Smart Older Adult Care vocational students, not for nursing students or registered nurses.

### 3.2 Expert positivity and authority

Expert positivity was evaluated by the response rate of the Delphi surveys. In the first Delphi round, 18 questionnaires were distributed and 17 were returned, yielding a response rate of 94.4%. Thirteen experts (76.47%) provided constructive comments to refine the preliminary evaluation framework. In the second round, all 17 distributed questionnaires were returned, with 3 experts (17.65%) providing minor comments. The authority coefficient (*Cr*), which was calculated as the average of the familiarity coefficient (*Cs*) and the judgment basis coefficient (*Ca*). The mean *Cs* for the experts was 0.765, and the mean *Ca* was 0.918, resulting in an overall *Cr* of 0.841 (*Cr* > 0.8), indicating the high authority of the expert panel and the reliability of their feedback.

### 3.3 Expert opinion coordination

The coordination of expert opinions was evaluated by the coefficient of variation (*CV*) and Kendall’s coefficient of concordance (*W*). In the first round, the *CV*s for individual indicators ranged from 0 to 0.217, all within the acceptable threshold of ≤ 0.25, indicating good consistency. In the second round, the *CV*s of all indicators further decreased, ranging from 0 to 0.115 (all ≤ 0.25), reflecting improved consistency after revision. Kendall’s *W* increased from 0.217 in the first round to 0.246 in the second, with both values statistically significant (*p* < 0.001), indicating a moderate but statistically significant consensus among experts. The improvement from Round 1 to Round 2 suggests that the iterative Delphi process, combined with controlled feedback, effectively moved expert opinions toward greater convergence. Detailed coordination coefficients for each level of the evaluation framework across the two Delphi rounds are shown in Table 2.

**Table 2:** Degree of coordination of expert opinions across Delphi rounds.

Level	First round				Second round			
	Index (*n*)	Kendall’s *W*	*χ* ^2^	*p*	Index (*n*)	Kendall’s *W*	*χ* ^2^	*p*
First-level	4	0.353	18.000	<0.001	4	0.353	18.000	<0.001
Second-level	14	0.134	29.646	0.005	13	0.203	41.333	<0.001
Third-level	46	0.237	185.708	<0.001	44	0.254	185.458	<0.001
Overall	64	0.217	236.550	<0.001	61	0.246	250.731	<0.001

### 3.4 Establishment of the framework

All items met the prespecified retention criteria in the first round, with mean scores ranging from 4.00 to 5.00, coefficients of variation (*CV*) from 0 to 0.22, and full-score rates from 52.9 to 100.0%. Drawing on expert comments and group discussion, modifications were made, including the addition of two third-level indicators, the deletion of one third-level indicator, the modification of 2 sec-level and two third-level indicators for improved clarity, and the merging of one pair of second-level indicators and three pairs of third-level indicators.

In the second round, all items again met the retention criteria, with mean scores of 4.47–5.00, *CV*s of 0.00–0.12, and full-score rates of 47.1–100.0%. Further adjustments were made based on experts’ feedback, including the addition of two third-level indicators, the consolidation of three third-level indicators related to online safety and privacy into one comprehensive indicator, and modification of one third-level indicator for enhanced clarity. Figure 1 provides a detailed summary of all modifications made across both Delphi rounds.

After the second round, expert consensus was achieved, resulting in a final evaluation framework consisting of 4 first-level, 13 s-level, and 44 third-level indicators. The complete framework with all indicators and weights is provided in Supplementary Table S1.

### 3.5 Indicator weights determined by AHP

The Analytic Hierarchy Process (AHP) was used to determine the weights of the indicators at all levels, with consistency testing performed to ensure the rationality of the weight distribution. AHP analysis showed that all consistency ratios (*CR*) of the judgment matrices at the first-level, second-level, and third-level were < 0.1, indicating satisfactory consistency. Table 3 presents a representative second-level judgment matrix (for the “Information Application and Innovation” dimension) with its consistency statistics, and Table 4 summarizes the consistency results for the first-level matrix.

**Table 3:** Example judgment matrix for the “Information Application and Innovation” dimension.

Second-level indicators	Information application ability	Information management ability	Information communication ability	Information innovation ability	Weight
Information application ability	1.0000	1.4765	2.6954	3.0672	0.4006
Information management ability	0.6773	1.0000	2.9716	3.4600	0.3482
Information communication ability	0.3710	0.3365	1.0000	1.9829	0.1512
Information innovation ability	0.3260	0.2890	0.5043	1.0000	0.1001

This aggregated matrix was derived from 15 expert pairwise comparisons using the geometric mean method. Λmax = 4.0684, CI = 0.0228, RI = 0.89 (*n* = 4), CR = 0.0256 < 0.1, indicating satisfactory consistency.

**Table 4:** Consistency statistics for the first-level judgment matrix.

Matrix	*n*	*λ*_max_	*CI*	*RI*	*CR*
First-level indicators (4 dimensions)	4	4.0671	0.0224	0.89	0.0252

The first-level indicators ranked by weight as follows: Information Application and Innovation (0.4718) > Information Awareness (0.3073) > Information Knowledge (0.1228) > Information Ethics and Security (0.0981). This ranking reflects the emphasis on practical application and innovation abilities in information literacy for higher vocational students majoring in Smart Older Adult Care, which is consistent with the talent cultivation objectives of the major and the practical demands of the smart older adult care industry. Detailed consistency results for all judgment matrices—including λmax, *CI*, *RI*, and *CR* for each matrix—along with the corresponding weight vectors, are provided in Supplementary Table S2. The complete pairwise comparison matrices are available from the corresponding author upon reasonable request.

## 4 Discussion

This study developed an information literacy evaluation framework for higher vocational students in Smart Older Adult Care. The resulting framework comprises four dimensions covering awareness, knowledge, application/innovation, and ethics/security. It is structured to move from basic capabilities toward higher-order skills, which directly addresses a common problem: generic information literacy frameworks often do not fit the specific needs of vocational training (23). For instance, our framework includes items about using smart monitoring devices and protecting older adults’ personal information. Such items link general information literacy with professional practice, something also emphasized by Dai et al. (8).

Compared with existing frameworks, our work offers several distinctive contributions. The ACRL Framework and ANZIIL Framework, while foundational, are designed for general higher education and lack literacy relevant to vocational disciplines. In contrast, our framework incorporates indicators directly relevant to smart older adult care practice, such as smart device operation in older adult care settings, data-informed care planning, and age-appropriate information communication. Unlike nursing informatics frameworks, which center on clinical nursing and evidence-based practice for registered nurses, our framework covers a wider workforce: older adult care providers and managers who, while not holding nursing licensure, need information skills to offer technology-enhanced care in various older adult care contexts. By integrating general information literacy with vocational education principles and smart older adult care context, our framework fills a gap not addressed by existing instruments.

### 4.1 The primacy of application and innovation

Information application and innovation was the most heavily weighted dimension. This outcome is consistent with the philosophy of vocational education, which emphasizes practical performance over theoretical knowledge (24). In smart older adult care, technology supports service delivery, so students must be able to translate data and digital tools into tangible care improvements. Xu et al. (25) similarly argued that older adult care professionals need stronger information technology (IT) application skills within the smart care model.

The emphasis on application is well documented in the literature. Competency-based education defines competence as demonstrable performance in authentic contexts, and digital health competency models similarly prioritize practical technology skills (26, 27). However, our findings extend beyond these frameworks. While most existing models focus on the use of established tools, our results identify innovation as a distinct and valued sub-dimension. This distinction is significant: the Organization of Economic Co-operation and Development’s (OECD) Learning Compass 2030 identifies “creating new value” as a core competency (28), and Song et al. (29) found innovation essential for smart care professionals. Together, these observations suggest that employers value not only problem-solvers but also practitioners capable of generating novel responses to evolving care demands.

Within this dimension, information application ability and information management ability received the highest ratings, while innovation ability emerged as a separate sub-dimension. This pattern reflects a broader shift in educational expectations: using information is no longer sufficient; creating value from it has become equally important. Training programs should therefore move beyond routine operations. Simulated or project-based tasks, such as designing an intervention for cognitive decline, may help cultivate genuine innovative capacity.

### 4.2 The foundational role of information awareness

Information awareness ranked second, confirming its foundational status. What stood out was that the proactive willingness to use information was rated higher than merely perceiving its existence. This aligns with the ACRL framework’s threshold concepts of “scholarship as conversation” and “research as inquiry” (5), which encourage learners to move from passive reception to active knowledge construction. The high rating of “willingness to actively innovate services” further emphasizes an action-oriented mindset.

One might assume that “improvement awareness” is of lesser importance given its lower relative weight, but such interpretation would be misleading. The indicator “actively acquiring new knowledge and skills” (which falls under improvement awareness) had a very high local weight. In a fast-changing field like smart older adult care, continuous learning is indispensable. Jiang (30) made a similar observation, arguing that vocational education must cultivate the “ability to learn within the work process.” Awareness-building activities, including regular technology trend seminars, should be embedded throughout the curriculum rather than left as an abstract concept.

### 4.3 Knowledge in a vocational context

Information knowledge ranked third, but the more interesting finding concerns which types of knowledge matter. Retrieval knowledge and evaluation knowledge were consistently rated higher than broad theoretical knowledge. This reflects the task-driven nature of vocational education, where students need to locate and judge information directly relevant to their work. In contrast, general higher education may place greater value on theoretical breadth. Moodie (31) shows that practical know-how only pays off when tied to a specific work context, while broad theory often stays too abstract to use. That is why a well-built search strategy will serve our students better than a memorized list of IoT principles.

This finding supports case-based or problem-based teaching. For instance, asking students to develop a personalized nutrition plan for a frail older adult forces them to retrieve, evaluate and apply information. That said, foundational theory should not be neglected. Basic concepts are still important for higher-order skills. A practical way forward is to offer theoretical content just in time, such as through mini-lectures or knowledge maps in response to particular learning needs (32).

### 4.4 Ethics and security: low weight but non-negotiable

Information ethics and security received the lowest overall weight. This finding may be understood through the distinction between threshold and differentiating competencies (33, 34). Threshold competencies are baseline requirements expected of all practitioners and, while essential, do not differentiate superior from average performers. Differentiating competencies, by contrast, are those that set excellent performance from satisfactory practice. Experts in our Delphi study may have treated ethical behavior and security awareness as threshold competencies—automatic prerequisites for professional practice—rather than as differentiating variables. Similar patterns have been observed in other Delphi-AHP studies in health professions education (35). Importantly, low weight does not indicate low importance. The Quintuple Aim framework similarly prioritizes privacy awareness, ethical considerations, and data security (10), and World Health Organization (WHO) guidance on artificial intelligence (AI) ethics states that security controls and ethical principles are non-negotiable in any health data system (36). Thus, while ethics received a relatively modest weight for differentiation purposes, it remains a fundamental requirement for competent practice.

Within this domain, information security was consistently seen as the most critical part, with strong emphasis on respecting privacy and knowing what to do in case of a data breach. These are not optional extras but basic safeguards. Both ISO (International Organization for Standardization) 27,001 (37) and the WHO guidance on AI ethics (36) state clearly that security controls and ethical principles are non-negotiable in any health data system. Therefore, when using this framework for assessment, we recommend a gate-keeping rule: failing a critical ethical item—for example, accessing an older adult’s health record without consent—should automatically fail the entire assessment, regardless of scores in other dimensions.

### 4.5 Implications for education

The framework has direct implications for curriculum design in higher vocational programs that train students for smart older adult care roles. Rather than treating the dimensions as content areas to be covered, educators can use them as a structuring device for learning objectives. To build application and innovation skills, projects that require students to design and test technology-based care solutions are worth adopting. The gate-keeping role of ethics also means assessment systems need mandatory critical items; a failing mark on privacy violations should not be compensated by high scores elsewhere.

### 4.6 Practical feasibility

With 44 third-level indicators, the framework is comprehensive but may also pose practical challenges in implementation. Administrators should therefore weigh the time and resources required for full-scale use. Institutions may choose to administer the framework for periodic comprehensive evaluations (e.g., annual or semester-end assessments) rather than routine testing, or select subsets of indicators relevant to specific learning modules or training phases. The development of a web-based or mobile assessment platform could further reduce administrative burden by automating data collection and scoring. Future research should also develop a shortened version using empirical methods such as factor analysis or item response theory, so as to identify the most parsimonious yet informative set of indicators and thereby enhance the framework’s utility for regular formative use.

## 5 Limitations and future research

Several limitations should be acknowledged. Although geographically diverse, our expert panel may not fully capture perspectives from other cultural or educational contexts. The sample size (*n* = 17) was relatively modest, with some attrition during the AHP weighting stage. The framework has not yet been empirically validated, so its performance with actual student populations remains unknown. In addition, while the Delphi process provides evidence of content validity, we did not calculate supplementary validity indices because our 5-point Likert scale was not designed to generate the dichotomous relevance ratings required for those analyzes.

The moderate Kendall’s *W* values (0.217 and 0.246) suggest modest agreement, likely reflecting the diverse disciplinary backgrounds of the panel rather than a lack of consensus validity. The improvement from Round 1 to Round 2 indicates that the iterative process moved expert opinions toward greater convergence.

Future research will operationalize the framework into a formal questionnaire and conduct psychometric testing, including construct validity, criterion validity, reliability, and responsiveness assessments in student populations.

## 6 Conclusion

Guided by the DCAT, this study developed an expert-validated information literacy evaluation framework specifically for higher vocational students majoring in Smart Older Adult Care. The high weight on “Information Application and Innovation” underscores the vocational emphasis on practice, while the specific indicators capture the unique demands of the smart older adult care context. This evaluation framework can serve as a valuable tool for educators to assess student competencies, identify learning gaps, and guide curriculum development to better prepare future professionals for the digital transformation of older adult care. Further empirical validation of the system as a measurement instrument is the essential next step.

## Data Availability

The raw data supporting the conclusions of this article will be made available by the authors, without undue reservation.
