# Supplementary material for: Developing an information literacy competency evaluation framework for Chinese higher vocational students in smart older adult care: a Delphi-AHP study
Source: Front Public Health. 2026 Jul 29;14:1906092. doi: 10.3389/fpubh.2026.1906092 (PMC13461313; doi:10.3389/fpubh.2026.1906092)
Supplement: Supplementary file 1 [file Table_1.DOCX]

**Supplementary Table S1**

**Final Information Literacy Evaluation Framework with Weights**

| **Indicator** | **Importance Score**  **(*M*± *SD*)** | ***CV*** | **Full-score Rate (%)** | **Weight** | **Combination Weight** |
| --- | --- | --- | --- | --- | --- |
| **Ⅰ Information Awareness** | 5.00±0.00 | 0.00 | 100.00 | 0.3073 | - |
| ***Ⅰ-1 Information Perception Awareness*** | 5.00±0.00 | 0.00 | 100.00 | 0.3515 | 0.1080 |
| Ⅰ-1-1 Recognizes the critical role of information in senior care services and actively gathers relevant data. | 4.94±0.24 | 0.05 | 94.10 | 0.4009 | 0.0433 |
| Ⅰ-1-2 When faced with challenges in care, can quickly identify and connect to potentially useful information resources. | 5.00±0.00 | 0.00 | 100.00 | 0.3501 | 0.0378 |
| Ⅰ-1-3 Keeps abreast of new technologies in the smart senior care field and proactively assesses their potential value for service improvement. | 5.00±0.00 | 0.00 | 100.00 | 0.2490 | 0.0269 |
| ***Ⅰ-2 Information Application Awareness*** | 5.00±0.00 | 0.00 | 100.00 | 0.4003 | 0.1230 |
| Ⅰ-2-1 Recognizes the role of information in improving the quality of life of older adults and actively uses information technology to optimize services. | 4.82±0.39 | 0.08 | 82.40 | 0.2986 | 0.0367 |
| Ⅰ-2-2 Willing to use information technology to provide personalized service plans for older adults, such as customized health monitoring schedules. | 5.00±0.00 | 0.00 | 100.00 | 0.2989 | 0.0368 |
| Ⅰ-2-3 Demonstrates a willingness to innovate services by integrating technology into various care scenarios. | 5.00±0.00 | 0.00 | 100.00 | 0.4024 | 0.0495 |
| ***Ⅰ-3 Information Improvement Awareness*** | 5.00±0.00 | 0.00 | 100.00 | 0.2483 | 0.0763 |
| Ⅰ-3-1 Can follow and select key information such as policies, regulations, and cutting-edge technologies in smart elderly care, understanding their potential impact on the profession. | 5.00±0.00 | 0.00 | 100.00 | 0.3971 | 0.0303 |
| Ⅰ-3-2 Can actively acquire new knowledge and skills through various means and apply them initially to analyze and solve practical problems in elderly care. | 5.00±0.00 | 0.00 | 100.00 | 0.6029 | 0.0460 |
| **Ⅱ Information Knowledge** | 5.00±0.00 | 0.00 | 100.00 | 0.1228 | - |
| ***Ⅱ-1 Theoretical Knowledge*** | 5.00±0.00 | 0.00 | 100.00 | 0.2476 | 0.0304 |
| Ⅱ-1-1 Can explain basic concepts such as information, data, and knowledge and their value in smart elderly care. | 5.00±0.00 | 0.00 | 100.00 | 0.3511 | 0.0107 |
| Ⅱ-1-2 Familiar with common types of information and their characteristics in elderly care services. | 5.00±0.00 | 0.00 | 100.00 | 0.2973 | 0.0090 |
| Ⅱ-1-3 Understands the typical applications and functions of technologies (e.g., IoT, big data, cloud computing) in senior care services. | 4.88±0.49 | 0.10 | 94.10 | 0.3516 | 0.0107 |
| ***Ⅱ-2 Information Retrieval Knowledge*** | 4.94±0.24 | 0.00 | 94.10 | 0.4020 | 0.0493 |
| Ⅱ-2-1 Can proficiently operate mainstream search engines and professional databases (e.g., CNKI, Wanfang, PubMed) to find literature and data related to elderly care. | 5.00±0.00 | 0.00 | 100.00 | 0.5531 | 0.0273 |
| Ⅱ-2-2 Can construct and revise effective search strategies based on information needs. | 5.00±0.00 | 0.00 | 100.00 | 0.4469 | 0.0221 |
| ***Ⅱ-3 Information Evaluation Knowledge*** | 5.00±0.00 | 0.00 | 100.00 | 0.3504 | 0.0430 |
| Ⅱ-3-1 Masters core criteria for evaluating information credibility (e.g., authority, accuracy, timeliness, objectivity). | 4.94±0.24 | 0.05 | 94.10 | 0.6023 | 0.0259 |
| Ⅱ-3-2 **Understands common strategies for ensuring information reliability, such as cross-referencing and source verification.** | 4.94±0.24 | 0.05 | 94.10 | 0.3977 | 0.0171 |
| **Ⅲ Information Application and Innovation** | 4.65±0.49 | 0.11 | 64.70 | 0.4718 | - |
| ***Ⅲ-1 Information Application Ability*** | 4.88±0.33 | 0.07 | 88.20 | 0.4006 | 0.1890 |
| Ⅲ-1-1 Can utilize tools like big data, cloud computing, and generative AI to find, filter, and judge information. | 5.00±0.00 | 0.00 | 100.00 | 0.1994 | 0.0377 |
| Ⅲ-1-2 Proficient in using office software for document editing, data processing, and information presentation to efficiently complete daily tasks. | 5.00±0.00 | 0.00 | 100.00 | 0.3040 | 0.0575 |
| Ⅲ-1-3 **Can operate smart devices to collect health data from older adults and perform preliminary analysis.** | 4.88±0.33 | 0.07 | 88.20 | 0.2457 | 0.0464 |
| Ⅲ-1-4 Masters the operation methods of common smart elderly care devices and can apply them proficiently in practical services. | 4.65±0.49 | 0.11 | 64.70 | 0.1475 | 0.0279 |
| Ⅲ-1-5 **When encountering technical problems, can find solutions by consulting relevant literature or subject-matter experts.** | 5.00±0.00 | 0.00 | 100.00 | 0.1034 | 0.0195 |
| ***Ⅲ-2 Information Management Ability*** | 4.76±0.44 | 0.09 | 76.50 | 0.3482 | 0.1643 |
| Ⅲ-2-1 Can integrate information like health data of older adults to provide a basis for developing personalized care plans. | 4.94±0.24 | 0.05 | 94.10 | 0.0964 | 0.0158 |
| Ⅲ-2-2 Can effectively filter, categorize, and summarize elderly care information, extracting key content to support decision-making. | 4.82±0.53 | 0.11 | 88.20 | 0.1297 | 0.0213 |
| Ⅲ-2-3 Can use data analysis tools for descriptive analysis and visualization of elderly care service operation data. | 4.94±0.24 | 0.05 | 94.10 | 0.1331 | 0.0219 |
| Ⅲ-2-4 Can use tools like shared documents and project management systems for collaborative information creation and management within a team. | 5.00±0.00 | 0.00 | 100.00 | 0.1847 | 0.0303 |
| Ⅲ-2-5 Can critically evaluate information (especially content generated by AI) using information evaluation criteria. | 4.94±0.24 | 0.05 | 94.10 | 0.1779 | 0.0292 |
| Ⅲ-2-6 Can use data analysis to identify problems in elderly care services and propose reasonable solutions. | 5.00±0.00 | 0.00 | 100.00 | 0.2782 | 0.0457 |
| ***Ⅲ-3 Information Communication Ability*** | 4.94±0.24 | 0.05 | 94.10 | 0.1512 | 0.0713 |
| Ⅲ-3-1 **Can effectively convey professional information in various formats, tailored to the cognitive needs of different audiences (e.g., older adults, colleagues).** | 5.00±0.00 | 0.00 | 100.00 | 0.3526 | 0.0252 |
| Ⅲ-3-2 Can use communication tools for daily interaction to ensure the smooth flow of elderly care service workflows. | 4.94±0.24 | 0.05 | 94.10 | 0.2959 | 0.0211 |
| Ⅲ-3-3 Can assess the digital literacy level of older adults and develop individualized teaching plans to bridge the digital divide. | 5.00±0.00 | 0.00 | 100.00 | 0.1479 | 0.0106 |
| Ⅲ-3-4 Can proficiently use tools like video conferencing and shared documents for remote collaboration. | 4.88±0.33 | 0.07 | 88.20 | 0.2036 | 0.0145 |
| ***Ⅲ-4 Information Innovation Ability*** | 4.65±0.49 | 0.11 | 64.70 | 0.1001 | 0.0472 |
| Ⅲ-4-1 Can actively explore potential application scenarios of cutting-edge information technology in smart elderly care and innovate service models. | 4.88±0.33 | 0.07 | 88.20 | 0.2537 | 0.0120 |
| Ⅲ-4-2 Can participate in innovative projects, using collected information to develop new solutions for elderly care practice. | 5.00±0.00 | 0.00 | 100.00 | 0.4001 | 0.0189 |
| Ⅲ-4-3 Can integrate multidisciplinary knowledge for innovative analysis and application of elderly care information resources. | 4.47±0.51 | 0.12 | 47.10 | 0.1975 | 0.0093 |
| Ⅲ-4-4 **When faced with complex problems, can use information technology for simulation and predictive analysis to inform decision-making.** | 4.59±0.51 | 0.11 | 58.80 | 0.1487 | 0.0070 |
| **Ⅳ Information Ethics and Security** | 5.00±0.00 | 0.00 | 100.00 | 0.0981 | - |
| ***Ⅳ-1 Information Ethics and Morality*** | 5.00±0.00 | 0.00 | 100.00 | 0.2512 | 0.0246 |
| Ⅳ-1-1 Adheres to information ethics in geriatric care, respects and protects intellectual property, and complies with academic norms. | 5.00±0.00 | 0.00 | 100.00 | 0.1968 | 0.0048 |
| Ⅳ-1-2 Obtains informed consent when using information and respects the dignity of older adults throughout the information processing lifecycle. | 5.00±0.00 | 0.00 | 100.00 | 0.4522 | 0.0111 |
| Ⅳ-1-3 Can recognize data bias and strives for fairness and non-discrimination in service decisions. | 5.00±0.00 | 0.00 | 100.00 | 0.3510 | 0.0086 |
| ***Ⅳ-2 Information Laws and Regulations*** | 5.00±0.00 | 0.00 | 100.00 | 0.2974 | 0.0292 |
| Ⅳ-2-1 **Familiar with relevant data privacy laws and regulations (e.g., Personal Information Protection Law)** | 5.00±0.00 | 0.00 | 100.00 | 0.4031 | 0.0118 |
| Ⅳ-2-2 Strictly adheres to relevant laws and regulations in elderly care practice and standardizes information processing procedures. | 5.00±0.00 | 0.00 | 100.00 | 0.5969 | 0.0174 |
| ***Ⅳ-3 Information Security*** | 5.00±0.00 | 0.00 | 100.00 | 0.4513 | 0.0443 |
| Ⅳ-3-1 Understands common information security threats and knows basic protective measures. | 5.00±0.00 | 0.00 | 100.00 | 0.1507 | 0.0067 |
| Ⅳ-3-2 When using smart devices, can take measures to ensure the secure storage and transmission of information. | 5.00±0.00 | 0.00 | 100.00 | 0.2030 | 0.0090 |
| Ⅳ-3-3 Can identify and prevent online fraud and actively help older adults resist harmful information. | 5.00±0.00 | 0.00 | 100.00 | 0.2495 | 0.0110 |
| Ⅳ-3-4 Has a strong sense of privacy protection and strictly adheres to information confidentiality principles. | 5.00±0.00 | 0.00 | 100.00 | 0.0991 | 0.0044 |
| Ⅳ-3-5 **Familiar with emergency plans and reporting procedures for data breaches, and can take immediate steps to mitigate risks.** | 5.00±0.00 | 0.00 | 100.00 | 0.2977 | 0.0132 |
