# Supplementary material for: Developing an information literacy competency evaluation framework for Chinese higher vocational students in smart older adult care: a Delphi-AHP study
Source: Front Public Health. 2026 Jul 29;14:1906092. doi: 10.3389/fpubh.2026.1906092 (PMC13461313; doi:10.3389/fpubh.2026.1906092)
Supplement: Supplementary file 2 [file Table_2.DOCX]

**Supplementary Table S2**

**Summary Consistency Results and Weight Vectors for all Judgment Matrices**

| **Matrix Level** | **Matrix Name** | ***n*** | ***λ*max** | ***CI*** | ***RI*** | ***CR*** | **Weight Vector** |
| --- | --- | --- | --- | --- | --- | --- | --- |
| **First-level** | All 4 dimensions | 4 | 4.0671 | 0.0224 | 0.89 | 0.0252 | (0.3073, 0.1228, 0.4718, 0.0981) |
| **Second-level** | I. Information Awareness | 3 | 3.0119 | 0.0059 | 0.52 | 0.0113 | (0.3515, 0.4003, 0.2483) |
| **Second-level** | II. Information Knowledge | 3 | 3.0139 | 0.0070 | 0.52 | 0.0135 | (0.2476, 0.4020, 0.3504) |
| **Second-level** | III. Information Application and Innovation | 4 | 4.0684 | 0.0228 | 0.89 | 0.0256 | (0.4006, 0.3482, 0.1512, 0.1001) |
| **Second-level** | IV. Information Ethics and Security | 3 | 3.0218 | 0.0109 | 0.52 | 0.0210 | (0.2512, 0.2974, 0.4513) |
| **Third-level** | I-1. Information Perception Awareness (3 indicators) | 3 | 3.0144 | 0.0072 | 0.52 | 0.0138 | (0.4009, 0.3501, 0.2490) |
| **Third-level** | I-2. Information Application Awareness (3 indicators) | 3 | 3.0161 | 0.0080 | 0.52 | 0.0154 | (0.2986, 0.2989, 0.4024) |
| **Third-level** | I-3. Information Improvement Awareness (2 indicators) | 2 | 2.0001 | 0.0001 | 0.00 | — | (0.3971, 0.6029) |
| **Third-level** | II-1. Information Theoretical Knowledge (3 indicators) | 3 | 3.0161 | 0.0080 | 0.52 | 0.0154 | (0.3511, 0.2973, 0.3516) |
| **Third-level** | II-2. Information Retrieval Knowledge (2 indicators) | 2 | 2.0000 | 0.0000 | 0.00 | — | (0.5531, 0.4469) |
| **Third-level** | II-3. Information Evaluation Knowledge (2 indicators) | 2 | 2.0000 | 0.0000 | 0.00 | — | (0.6023, 0.3977) |
| **Third-level** | III-1. Information Application Ability (5 indicators) | 5 | 5.0891 | 0.0223 | 1.12 | 0.0199 | (0.1994, 0.3040, 0.2457, 0.1475, 0.1034) |
| **Third-level** | III-2. Information Management Ability (6 indicators) | 6 | 6.1012 | 0.0202 | 1.26 | 0.0160 | (0.0964, 0.1297, 0.1331, 0.1847, 0.1779, 0.2782) |
| **Third-level** | III-3. Information Communication Ability (4 indicators) | 4 | 4.0340 | 0.0113 | 0.89 | 0.0127 | (0.3526, 0.2959, 0.1479, 0.2036) |
| **Third-level** | III-4. Information Innovation Ability (4 indicators) | 4 | 4.0475 | 0.0158 | 0.89 | 0.0178 | (0.2537, 0.4001, 0.1975, 0.1487) |
| **Third-level** | IV-1. Information Ethics and Morality (3 indicators) | 3 | 3.0120 | 0.0060 | 0.52 | 0.0115 | (0.1968, 0.4522, 0.3510) |
| **Third-level** | IV-2. Information Laws and Regulations (2 indicators) | 2 | 2.0000 | 0.0000 | 0.00 | — | (0.4031, 0.5969) |
| **Third-level** | IV-3. Information Security (5 indicators) | 5 | 5.0820 | 0.0205 | 1.12 | 0.0183 | (0.1507, 0.2030, 0.2495, 0.0991, 0.2977) |

*Note: All CR values were below 0.1, indicating acceptable consistency across all judgment matrices. RI values were taken from Saaty's random index table. The complete pairwise comparison matrices are not displayed in this table; refer to the original AHP computation files for full matrix data.*
